# A High-Precision Temperature Compensation Method for TMR Weak Current Sensors Based on FPGA

**DOI:** 10.3390/mi15121407

**Published:** 2024-11-22

**Authors:** Jie Wu, Ke Zhou, Qingren Jin, Baihua Lu, Zhenhu Jin, Jiamin Chen

**Affiliations:** 1 State Key Laboratory of Transducer Technology, Aerospace Information Research Institute, Chinese Academy of Sciences, Beijing 100190, China; 2 School of Electronic, Electrical and Communication Engineering, University of Chinese Academy of Sciences, Beijing 100049, China; 3 Electric Power Research Institute of Guangxi Power Grid Co., Ltd., Nanning 530023, China; 4 College of Materials Sciences and Opto-Electronic Technology, University of Chinese Academy of Sciences, Beijing 100049, China

**Keywords:** magnetic sensor, TMR current sensor, leakage current, software temperature compensation, FPGA

## Abstract

Tunnel magnetoresistance (TMR) sensors, known for their high sensitivity, efficiency, and compact size, are ideal for detecting weak currents, particularly leakage currents in smart grids. However, temperature variations can negatively impact their accuracy. This work investigates the effects of temperature variations on measurement accuracy. We analyzed the operating principles and temperature characteristics of TMR sensors and proposed a high-precision, software-based temperature compensation method using cubic spline interpolation combined with polynomial regression and zero-point self-calibration. Additionally, a field-programmable gate array (FPGA)-based temperature compensation circuit was designed and implemented. An experimental platform was established to comprehensively evaluate the sensor’s performance under various temperature conditions. Experimental results demonstrate that this method significantly enhances the sensor’s temperature stability, reduces the sensitivity temperature drift coefficient, and improves zero-point drift stability, outperforming other compensation methods. After compensation, the sensor’s measurement accuracy in complex temperature environments is substantially improved, enabling effective weak current detection in smart grids across diverse environments.

## 1. Introduction

The growing demand for safety, stability, and energy efficiency has driven the development of smart grids [1,2,3]. Precise current measurement and monitoring are critical for the efficient management, fault detection, and dynamic optimization of smart grids, particularly in the context of leakage current detection [4,5]. Surge arresters are used to protect various electrical devices within power systems from damage caused by over-voltages. When a surge arrester is in good condition, a current ranging from tens to hundreds of microamperes flows through it at normal operating voltages. However, prolonged operation can lead to the aging of resistive elements, and humid environments can adversely affect insulation properties, resulting in the degradation of the surge arrester’s performance. In severe cases, this degradation may even cause explosive accidents. Under such conditions, the leakage current of the surge arrester increases toseveral hundred microamperes to several milliamperes and spanning frequencies from DC increase toseveral tens of Hertz [6,7,8,9]. By using a current sensor with a measurement range of ±10 mA to monitor the changes in leakage current, surge arrester faults can be promptly detected, thereby ensuring the safe operation of the power grid. Therefore, the development of sensors capable of detecting low-frequency, weak currents is essential for ensuring the stability and reliability of power systems.

In recent years, tunneling magnetoresistance (TMR) sensors have demonstrated significant potential in current measurement applications due to their high sensitivity, low noise, excellent linearity, and broad frequency response. These characteristics make TMR sensors particularly suitable for detecting leakage currents [10,11,12]. Furthermore, the small size and low power consumption of TMR sensors enable their cost-effective and large-scale deployment in smart grids. In contrast, traditional current sensors such as current transformers (CTs) [13,14] are bulky, expensive, and incapable of measuring DC currents. Rogowski coils [15,16] are insensitive to DC and low-frequency signals and suffer from low precision, while fluxgate sensors [17,18] are complex, expensive, and susceptible to external magnetic field interference. Similarly, Hall sensors [19,20,21] have low sensitivity and accuracy, and their performance is significantly affected by temperature variations. Other magnetoresistance sensors, such as anisotropic magnetoresistance sensors (AMR) [22,23] and giant magnetoresistance sensors (GMR) [24,25,26], also exhibit limitations, including low sensitivity, poor linearity, and low accuracy. Given these challenges, TMR sensors offer a distinct advantage in measuring leakage currents in power grids, contributing significantly to the safe operation of smart grids.

Despite these advantages, TMR sensors are highly sensitive to temperature variations [27,28], which is particularly problematic in smart grid applications. Smart grids often cover vast geographical areas, where environmental temperatures can range from −40 °C to 50 °C. Moreover, TMR sensors installed near power lines or distribution cabinets may experience even higher temperatures, sometimes exceeding 100 °C, due to thermal radiation and self-heating. These extreme temperature fluctuations can severely compromise the accuracy of current measurements, presenting a major challenge for the widespread deployment of TMR sensors in smart grids. To ensure their reliable operation in such diverse conditions, addressing temperature drift and improving thermal stability are crucial for the practical application of TMR sensors. For TMR sensors to be viable for smart grid applications, they must maintain a stable output across a wide temperature range, ideally from −40 °C to 120 °C.

To mitigate temperature drift in this range, temperature compensation methods are applied to improve thermal stability [29,30,31]. These methods are generally classified as hardware and software compensation. Hardware compensation involves integrating components such as thermistors or diodes into the sensor’s interface circuit to counteract the sensor’s temperature coefficient and offset the impact of temperature on output. Conversely, software compensation relies on pre-testing the sensor’s temperature characteristics and employing algorithms to correct temperature-induced errors in real-time based on environmental conditions.

Previous studies have demonstrated various approaches to temperature compensation. In Reference [28], a thermistor is integrated into the interface circuit to control the TMR sensor’s supply voltage, ensuring the voltage changes inversely with sensitivity, reducing the temperature sensitivity coefficient from 1780 ppm/°C to 504 ppm/°C. Although the temperature sensitivity is reduced to a certain extent, it makes the circuit more complicated. In Reference [32], a temperature sensor is used to collect temperature signals and adjust the gain of an amplifier, reducing the temperature sensitivity coefficient of a GMR sensor from 2498 ppm/°C to 678 ppm/°C. In Reference [33], a similar method is applied to a GMR current sensor, reducing its output temperature dependence from 0.0738%/°C to 0.0319%/°C. This method requires not only computational processing but also additional hardware, which increases the complexity of the system. In Reference [34], least squares and particle swarm optimization algorithms are applied to giant magnetoresistance elements, achieving relative full-scale measurement errors of 0.13% and 0.14% within the −40 °C to 80 °C range, respectively. In Reference [35], least squares data fitting is applied to reduce the TMR sensor’s temperature coefficient from 1100 ppm/°C to 117 ppm/°C and zero-output drift from 67.7 nT to 7.37 nT within the −45° C to 85 °C range. Although the least squares method in these two studies has a simple compensation operation, the accuracy after compensation is not very high. In References [36,37], B-spline mathematical modeling for the temperature compensation of a GMR current sensor is employed, achieving measurement accuracy within ±0.6% over a 50A range. This method completes temperature compensation for large currents but has not yet been implemented in weak current scenarios. The authors of Reference [38] propose using a TMR sensor for the dual-parameter measurement of current and temperature, simultaneously performing temperature compensation and achieving a current measurement accuracy of ±2.03% within the −40 °C to 160 °C range. But its measurement accuracy still needs to be improved. Therefore, this study aims to explore a new temperature compensation strategy that can further improve the measurement accuracy of the TMR current sensor without increasing the complexity of the system.

Hardware compensation provides a quick response, independence, and high reliability, but is generally limited to analog sensors, has complex circuit designs, and offers relatively low precision [39,40]. In contrast, software compensation is characterized by higher precision, greater flexibility, broader applicability, and the ease of updates and iterations [41]. This approach allows for compensation over a wider temperature range without frequent circuit modifications, preventing material waste. As a result, software compensation is considered more advantageous for addressing temperature drift in TMR sensors.

This paper presents a high-precision software-based temperature compensation method for TMR weak current sensors. The proposed method combines cubic spline interpolation with polynomial regression algorithms to compensate for the sensor’s sensitivity temperature drift. Additionally, a self-calibrating circuit is designed to address zero-point temperature drift. By integrating the hardware circuit with the high-speed data processing capabilities of a field-programmable gate array (FPGA), real-time temperature compensation for both sensitivity and zero-point output is achieved. This significantly enhances the monitoring capabilities of TMR current sensors for leakage currents in complex temperature environments within smart grids, ensuring the safe and stable operation of smart grid systems. Compared to existing methods, the proposed approach offers a streamlined process, high efficiency, and optimal resource utilization, making it highly suitable for large-scale industrial sensor applications.

## 2. Preliminary Principle

### 2.1. Principle of TMR Current Sensor

**Figure 1:**
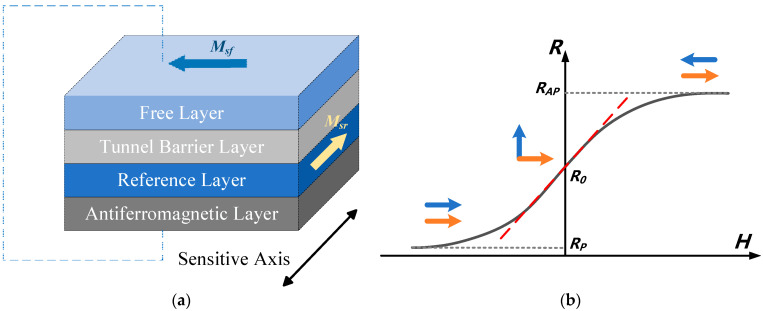
TMR structure and principle. (**a**) MTJ structure; (**b**) MTJ resistance as a function of magnetic field.

TMR arises from spin-dependent tunneling in a magnetic tunnel junction (MTJ) structure, comprising a free layer, tunnel barrier, and reference layer [42,43,44]. TMR resistance depends on the magnetic alignment between the free and reference layers. The alignment, parallel (low resistance) or antiparallel (high resistance), creates a linear relationship between magnetic field strength and resistance, which is critical for sensor applications, shown in Figure 1 [45,46,47].

**Figure 2:**
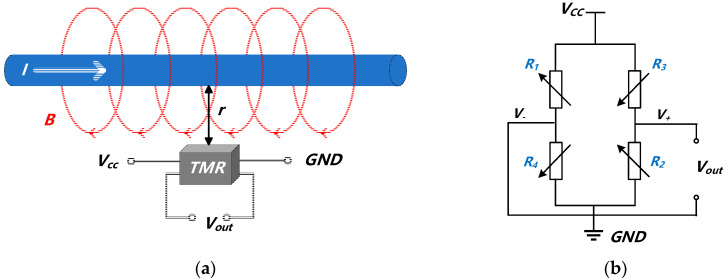
TMR current sensor. (**a**) Principle of TMR sensor current; (**b**) TMR Wheatstone bridge structure.

TMR current sensors use the TMR effect to detect magnetic fields from conductor currents. This generates proportional changes in magnetoresistance, as shown in Figure 2, which can be described by

(1) $$B=\mu_{0}I\;/2\pi r$$

(2) ∆R=αB

where *B* is the magnetic field strength generated by the current; *μ*_0_ is the vacuum permeability, which represents the characteristics of the magnetic field in a vacuum; *I* is the current passing through the conductor; *r* is the distance from the current to the measurement point. ∆R is the magnetoresistance change, and α is a proportionality coefficient related to the magnetic field. TMR sensors use a full-bridge Wheatstone configuration to enhance measurement accuracy, improve sensitivity, and reduce temperature effects [47,48,49,50]. The output is represented as

(3) $$\begin{array}{cc} V_{out} & =V_{out1}-V_{out2}\\ & =V_{cc}\cdot (\frac{R_{4}+\Delta R}{R_{1}-\Delta R+R_{4}+\Delta R}-\frac{R_{2}-\Delta R}{R_{3}+\Delta R+R_{2}-\Delta R})\\ & =V_{cc}\cdot \frac{\Delta R}{R}. \end{array}$$

Based on Equations (1) and (2), the relationship between the output and the current can be expressed as

(4) $$V_{out}=V_{cc}\cdot \frac{\mu_{0}\alpha }{2\pi rR}\cdot I=V_{cc}\cdot S\cdot I,$$

where *S* is the sensor sensitivity. Within the TMR devices’ linear range, the bridge output is proportional to the measured current, allowing direct determination of current magnitude from the measurement output.

**Figure 3:**
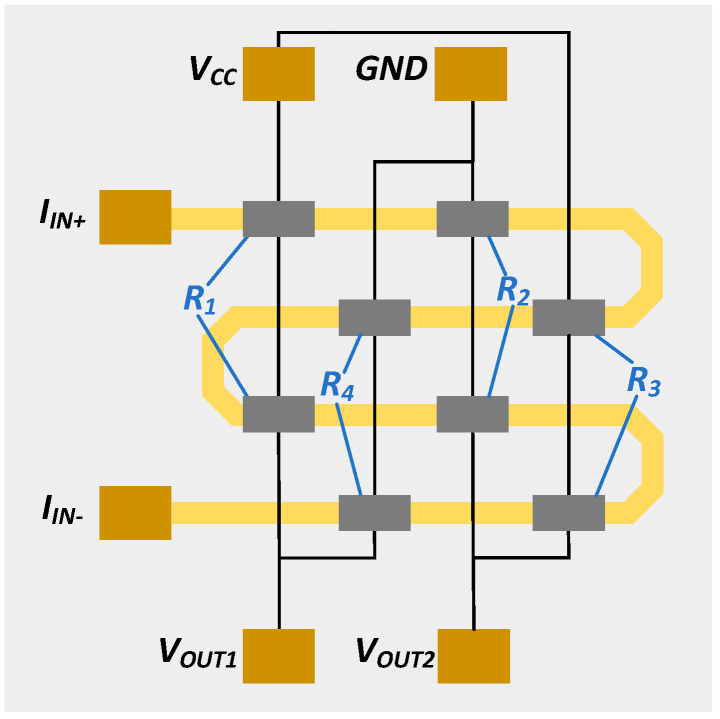
TMR current sensor microstructure.

TMR current sensor sensitivity can be optimized by adjusting the spacing between the conductor and the sensor element [45]. Through MEMS fabrication technology, the current conductor and TMR elements are integrated on one chip, reducing stray magnetic field interference and improving the signal–noise ratio, as shown in Figure 3. This approach enhances sensor sensitivity, achieves miniaturization, and controls costs, making it suitable for measuring weak currents. The magnetic field-sensitive chip used in this study to measure weak leakage currents is manufactured by MultiDimension Technology, Zhangjiagang, China, model TMR-MAC005. This chip integrates a current coil with a highly sensitive TMR element. By placing the TMR element in close proximity to the coil, it enables high-precision measurements of weak currents within the ±10 mA range. To enhance stability, multiple MTJs are connected in series in each bridge arm to prevent the adverse effects of low-yield MTJs on overall performance. In summary, the TMR chip achieves high precision and stable weak current detection through optimized design.

### 2.2. Temperature Characteristics

TMR element performance is significantly affected by temperature. Magnetic susceptibility and the magnetoresistance coefficient of magnetic materials change with temperature, influencing the sensor’s output signal. Temperature-induced resistance changes affect the stability and accuracy of the sensor’s output. Thermal noise (including thermal magnetic noise and thermal resistance noise) varies with temperature, affecting the element’s sensitivity and resolution. Additionally, temperature changes can cause materials to expand or contract, affecting the sensor’s structure and performance, thereby impacting measurement results [51,52]. In summary, temperature changes affect performance through multiple mechanisms. Effective temperature compensation is essential to mitigate these negative impacts and improve the sensor’s reliability and accuracy.

This paper analyzes the impact of temperature on TMR sensor performance from an application perspective, focusing on temperature characteristics based on Wheatstone bridge configuration. By introducing parameters *R_T_* and Δ*R_T_*, the impact of temperature changes on TMR elements is quantified. *R_T_* represents the temperature-dependent variation, while Δ*R_T_* indicates magnetoresistance change influenced by both temperature and the magnetic field, which can be expressed as

(5) $$\Delta R_{T}=\beta \left(T\right)\cdot B$$

Considering the temperature effect, the bridge output becomes

(6) $$V_{out}=V_{out1}-V_{out2}=V_{cc}\cdot \frac{\Delta R+\Delta R_{T}}{R+R_{T}}$$

According to Equations (1) and (5), the bridge output can be further expressed as

(7) $$V_{out}=V_{cc}\cdot \frac{[\alpha +\beta (T)]\cdot \mu_{0}}{2\pi r(R+R_{T})}\cdot I=V_{cc}\cdot S(T)\cdot I$$

It can be observed that temperature affects the voltage output of the sensor, resulting in sensitivity temperature drift.

The zero output of the TMR current sensor will drift due to temperature effects. Ideally, the four magnetoresistance elements in the bridge would be equally affected by temperature, resulting in zero output at the zero position. Due to manufacturing limitations, temperature coefficients do not match, and magnetoresistance elements are affected differently by temperature [53], leading to zero-output drift, meaning output is not zero when there is no magnetic field.

(8) $$\begin{array}{cc} V_{0} & =V_{cc}(\frac{R_{4}+R_{T4}}{\left(R_{1}+R_{T1}\right)+\left(R_{4}+R_{T4}\right)}-\frac{R_{2}+R_{T2}}{\left(R_{2}+R_{T2})+(R_{3}+R_{T3}\right)})\\ & \neq 0 \end{array}$$

**Figure 4:**
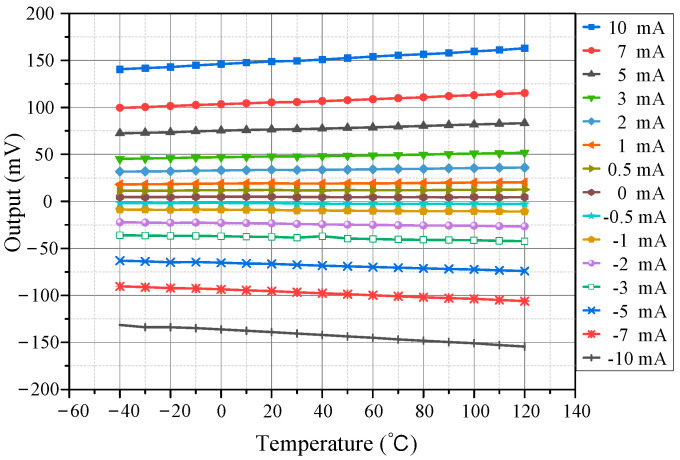
Sensor’s output at different temperatures.

After establishing the temperature characteristic model of the TMR sensor, the actual temperature characteristics were tested. First, the test chamber temperature was set to 20 °C with a bias voltage of 5 V. Multiple current test points within the ±10 mA range were selected and their outputs recorded. Then, the tests were repeated at 10 °C intervals from −40 °C to 120 °C. Results are shown in Figure 4.

**Figure 5:**
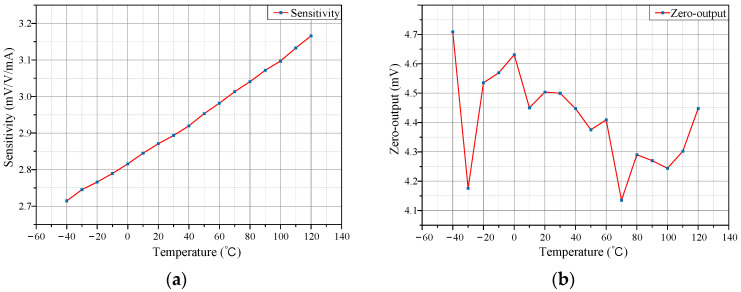
Temperature characteristics of TMR sensors. (**a**) Sensitivity curve; (**b**) Zero-output curve.

The sensitivity of the TMR current sensor is calculated according to Equation (9):

(9) $$k_{s}=\frac{k}{U_{s}}$$

where *k_s_* is the sensitivity (mV/V/mA), *k* is the slope of the best fit line between the output voltage and the measured current (mV/mA), and *U*_s_ is the supply voltage (V). The sensitivity–temperature characteristic curve of the TMR sensor is shown in Figure 5a. It can be seen that within the operating temperature range of −40 °C to 120 °C, the TMR current sensor exhibits significant sensitivity temperature drift. The zero-output temperature characteristic curve is shown in Figure 5b, indicating that the zero output deviates significantly from zero and fluctuates with temperature.

The temperature-dependent sensitivity and zero-output drift of TMR current sensors lead to significant measurement inaccuracies, particularly in environments with fluctuating temperatures [54]. Effective compensation strategies are essential to ensure precision and reliability.

## 3. Temperature Compensation Theory

### 3.1. Mathematical Model

According to the principle of the TMR current sensor, its output is determined by sensitivity, zero output, and input current, denoted by *S*, *V*_0_, *I* respectively, with *V_CC_* being the fixed bias voltage.

(10) $$V_{out}=V_{CC}\cdot S(T)\cdot I+V_{0}(T)$$

At the standard operating temperature *T*_0_, set to 20 °C, the sensitivity and zero output are denoted as *S*(*T*_0_) and *V*_0_(*T*_0_).

The zero output fluctuates significantly with temperature, following a complex pattern and showing poor repeatability at the same temperature. To address this issue, a zero-point self-calibration circuit was designed. This circuit utilizes an FPGA-controlled single pole double throw analog switch (SPDT) to obtain the zero-compensation coefficient *V*_0_*R*(*T*) on-site, thereby achieving real-time zero-temperature drift compensation. The temperature drift of sensitivity exhibits better repeatability and can be pre-measured. The characteristics are analyzed to obtain compensation coefficients, which are subsequently used for real-time compensation via a lookup table during measurement. The relationship between the temperature compensation coefficient for sensitivity and sensitivity is as follows:

(11) $$S_{R}(T)=\frac{S(T_{0})}{S(T)}$$

This paper proposes a temperature compensation algorithm that combines cubic spline interpolation with polynomial regression to derive sensitivity compensation coefficients across the temperature range of −40 to 120 °C. The temperature compensation is then performed according to Equation (12):

(12) $$\begin{array}{cc} V_{out}^{\prime } & =[V_{out}-V_{0R}(T)]\cdot S_{R}(T)\\ & =V_{CC}\cdot [S(T)\cdot S_{R}(T))]\cdot I\\ & =V_{CC}\cdot S(T_{0})\cdot I \end{array}$$

After compensation, the sensor output is proportional to the current, with a constant proportionality coefficient, ensuring stabilized sensitivity and thereby achieving precise measurements at any temperature.

### 3.2. Compensation Algorithm

This study employs a temperature compensation algorithm that integrates cubic spline interpolation with polynomial regression to derive sensitivity temperature compensation coefficients. Initially, cubic spline interpolation is used to estimate output data for additional sample points based on existing test data, thereby increasing the sample size and providing a foundation for polynomial regression. Cubic spline interpolation avoids the Runge phenomenon commonly associated with high-order interpolation and addresses endpoint non-smoothness found in standard piecewise interpolation methods. Polynomial regression models the sensitivity–temperature relationship using polynomial functions, capturing complex interactions and providing a precise fit over the temperature range of −40 °C to 120 °C. This combined approach results in high-precision temperature compensation coefficients.

Cubic spline interpolation is an effective piecewise method that constructs cubic polynomials for each segment based on a given set of sample points, approximating data behavior within each subinterval. The sensitivity–temperature relationship curve is constructed by partitioning the temperature interval [*T*_min_,*T*_max_] into *n* subintervals: [*T*_1_,*T*_2_], [*T*_2_,*T*_3_], … [*T_i_*,*T_i_*_+1_], … [*T_n_*,*T_n_*_+1_], where *T*_1_ = *T*_min_ and *T_n_*_+1_ = *T*_max_. For each subinterval, we perform piecewise interpolation on sensitivity, resulting in the function *S*(*t*), which must satisfy the following conditions:

1. *S*(*t*) is a piecewise function, and within each subinterval, it is a cubic function;
2. The interpolation conditions are met, ensuring equal function values at adjacent subinterval junctions;
3. The function has the same first and second derivatives at the connecting points of adjacent subintervals.

Based on these conditions, *S*(*t*) is derived to interpolate the sensitivity–temperature curve. This method estimates values between existing sample points, increasing the original 17 sample points to 33 sample points, which reduces the need for additional experimental data collection.

Polynomial regression is a method that models data points using polynomial functions. By introducing polynomial features, it enhances linear regression to better accommodate data variations. The polynomial regression model for temperature compensation is defined as follows:

(13) $$S=\beta_{0}+\beta_{1}t+\beta_{2}t^{2}+\beta_{3}t^{3}+\cdots +\beta_{n}t^{n}=\sum_{i=0}^{n}\beta_{i}t^{i}$$

where *S* represents sensitivity, *t* denotes temperature, *n* is the degree of the polynomial, and *β_i_* are the polynomial coefficients. The objective is to minimize the fitting error of the model, typically using the ordinary least squares (OLS) approach, which aims to minimize the following loss function:

(14) $$J(\beta )=\sum_{j=1}^{m}\;{\left(S_{j}^{\prime }-S_{j}\right)}^{2}=\sum_{j=1}^{m}\;{\left(\sum_{i=0}^{n}\beta_{i}t_{j}^{i}-S_{j}\right)}^{2}$$

where *S_j_*^′^ is the predicted sensitivity value, and *S_j_* is the actual sensitivity value at different test temperatures.

**Figure 6:**
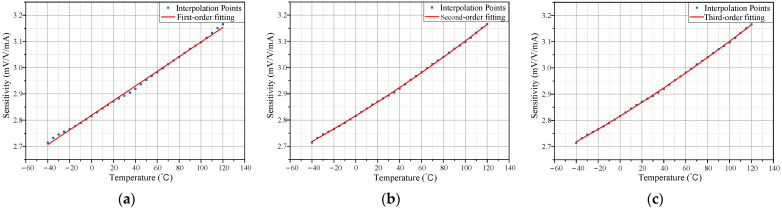
Results of polynomial regression for different degrees. (**a**) First-order; (**b**) Second-order; (**c**) Third-order.

A polynomial regression model is established based on existing temperature data, with the polynomial degree adjusted to optimize accuracy. For large datasets, a low polynomial degree results in fitting errors and poor compensation accuracy, while a high degree increases computation time and risks overfitting. This study compared the fitting performance of first-, second-, and third-order polynomial regressions, shown in Figure 6. The root mean square error (RMSE) of the fitting results for each degree was calculated using

(15) $$RMSE=\sqrt{\frac{1}{n}\sum_{i=1}^{n}\;{\left({\hat{S}}_{i}-S_{i}\right)}^{2}}$$

The smaller the RMSE value, the better the model accuracy. As shown in Table 1, the second-order and third-order regression models performed better than the first-order model. Considering that the second-order model requires fewer resources and achieves comparable accuracy, it was selected as the final approach.

### 3.3. System Structure

**Figure 7:**
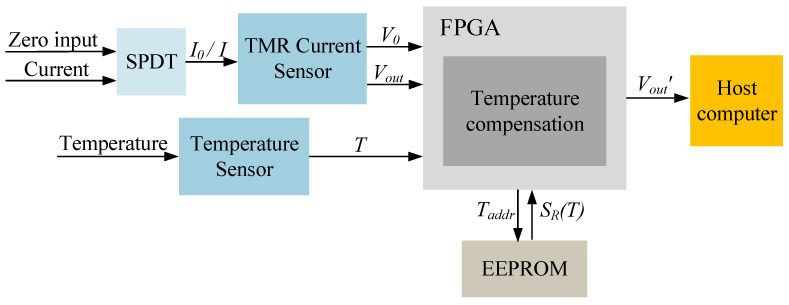
Structure of the temperature compensation system.

The temperature compensation system structure is depicted in Figure 7. The FPGA, serving as the system’s core controller, manages the SPDT circuit to perform zero-point temperature compensation. Sensitivity compensation coefficients are derived from cubic spline interpolation combined with a polynomial regression-based algorithm. The compensation process relies on data from a temperature sensor and EEPROM, with all calculations performed within the FPGA. The final compensated output is then transmitted to the host computer for further analysis.

## 4. Implementation

### 4.1. Hardware Circuit

**Figure 8:**
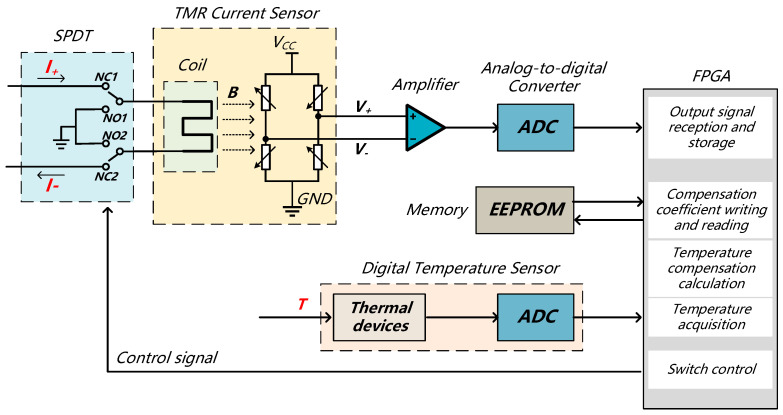
Schematic diagram of the hardware system structure.

Based on the proposed temperature compensation model, a TMR current sensor temperature compensation hardware circuit was designed. The structural diagram of the hardware circuit is depicted in Figure 8. The circuit performs two main functions: sensitivity temperature compensation and zero-output temperature compensation for the current sensor. The compensation process comprises two stages: online temperature calibration and real-time temperature. The circuit includes a TMR weak current sensor, an SPDT analog switch, a precision operational amplifier, an ADC, a DS18B20 temperature sensor, EEPROM, and an FPGA. Additionally, it incorporates auxiliary circuits for power regulation, signal transmission, and serial port output.

### 4.2. Temperature Compensation Process

The temperature calibration mode involves determining and storing the sensitivity temperature compensation coefficients of the TMR current sensor. Initially, temperature characteristics are tested, and the compensation coefficients are calculated using cubic spline and polynomial regression algorithms. These coefficients are then stored in the appropriate EEPROM memory addresses, corresponding to the operating temperature of the TMR sensor to ensure accurate addressing. Finally, the fixed-point compensation coefficients are sent to the FPGA via a serial port. The FPGA communicates with the EEPROM using the I2C protocol to write the coefficients into pre-allocated addresses for subsequent use during current measurement.

The current measurement mode achieves both current measurement and temperature compensation. Initially, the TMR current sensor measures the input current and outputs an initial voltage, which is significantly affected by ambient temperature. To correct the voltage, zero-point and sensitivity temperature compensation are applied. Zero-point temperature compensation relies on the SPDT circuit for self-calibration, managed by the FPGA. When the system is powered on, the FPGA sets the SPDT to the zero-input state (NO). In this state, the input current to the sensor is zero, and the FPGA records this output value as the zero-point compensation coefficient. Subsequently, the FPGA switches the SPDT to the normal measurement state (NC). The sensor measures the current under test, and the FPGA subtracts the zero-point compensation coefficient from the output value to complete the zero-point temperature compensation. Zero-point self-calibration is performed by the FPGA each time the system starts up. Sensitivity temperature compensation is performed in real time using the temperature sensor and EEPROM. During current measurement, the temperature sensor continuously monitors the ambient temperature. The FPGA reads the corresponding sensitivity compensation coefficients from the EEPROM and applies real-time compensation calculations. The compensated sensor output signal is uploaded to the host computer via the serial port, enabling precise current measurement.

## 5. Experimental Verification and Analysis

### 5.1. Compensation Effect Test

**Figure 9:**
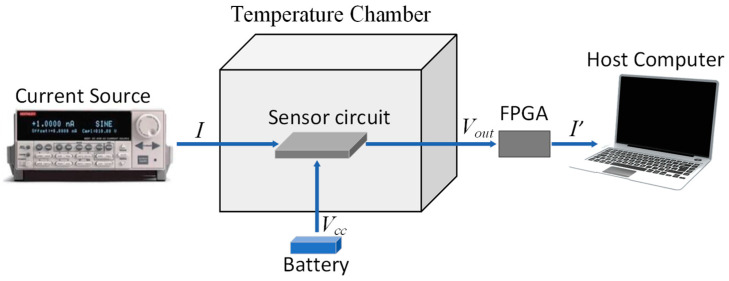
Experimental test platform.

An experimental test platform was constructed to verify the effectiveness of the temperature compensation approach for the TMR current sensor, as illustrated in Figure 9. The platform included an GSU-66V thermal chamber from ESPEC CORP., Osaka, Japan, providing an environmental temperature range of −60 °C to +150 °C, meeting the experimental requirements. The hardware system, with the temperature-compensated current sensor, was placed inside the thermal chamber and appropriately connected. The current under test was provided by a Keithley 6221 ultra-sensitive current source, capable of outputting high-precision AC and DC currents. A battery pack supplied power to the hardware circuit, providing the voltage bias for the TMR current sensor and power for the temperature sensor, EEPROM, and ADC. Communication between the host computer and the FPGA was established via an RS232 serial port.

**Figure 10:**
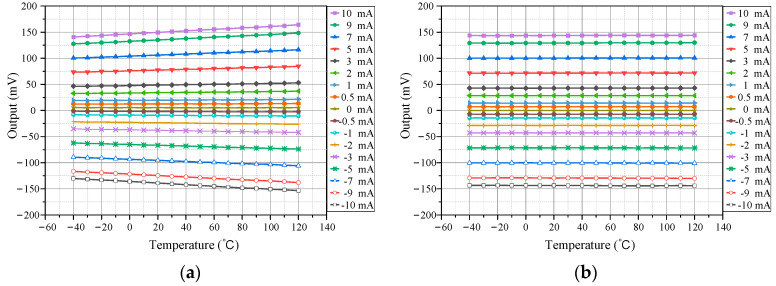
Sensor’s output before and after temperature compensation. (**a**) Output before compensation; (**b**) Output after compensation.

The differences in the TMR current sensor’s output before and after temperature compensation were first evaluated. Using the procedure described in Section 3, the thermal chamber temperature was set in the range of −40 °C to +120 °C, and the current range was set between −10 mA and +10 mA with a minimum step of 0.5 mA. Temperature data and the corresponding sensor system outputs, both before and after compensation, were recorded by the host computer. Figure 10 shows the current measurement results at various temperatures. The graph comparison indicates that after temperature compensation, the sensor’s output exhibited significantly reduced temperature-induced drift.

**Figure 11:**
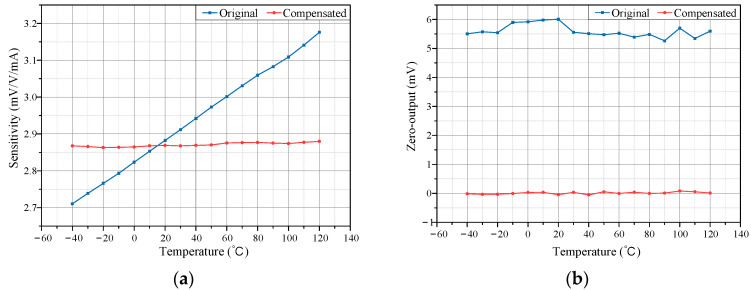
Comparison before and after compensation. (**a**) Comparison of sensitivity; (**b**) Comparison of zero-output.

To precisely assess sensitivity drift improvement resulting from temperature compensation, the sensor’s sensitivity at each temperature was calculated using Equation (9). Changes in sensitivity with temperature, both before and after compensation, are shown in Figure 11a. Results indicate that, prior to compensation, sensitivity exhibited significant variation with temperature, whereas post compensation, sensitivity remained stable across the temperature range.

To measure the impact of temperature changes on sensitivity, the temperature coefficient of sensitivity (TCS) is defined as follows:

(16) $$TCS=\frac{S_{max}-S_{min}}{S(T_{0})\times (T_{max}-T_{min})}\times 10^{6}\;ppm/{}^{\circ}C,$$

where *T*_max_ and *T*_min_ represent the maximum and minimum temperatures, while *S*_max_ and *S*_min_ denote the maximum and minimum sensitivities within the temperature range. A higher TCS value indicates a greater impact of temperature on sensor sensitivity, leading to larger measurement errors over a wide temperature range. According to Equation (16), the TCS without temperature compensation was calculated to be 1010 ppm/°C. After compensation, the TCS was reduced to 37 ppm/°C, representing a 96.34% reduction, and significantly enhancing the sensor’s sensitivity stability.

Figure 11b compares the zero output of the TMR current sensor before and after compensation. Before compensation, the average zero drift over the entire temperature range was 5.6025 mV, corresponding to a current measurement error of 0.39 mA. After compensation, the average zero drift decreased to 0.0090 mV, corresponding to a current measurement error of 0.62 µA, representing a 99.84% reduction in zero drift, meeting the accuracy requirements for leakage current measurement in power grids. The standard deviation of the zero-offset output further indicates the sensor’s zero-drift stability. Calculations show that the zero-drift stability improved from 0.2157 mV before compensation to 0.0370 mV after, marking an 82.85% improvement. Thus, zero-drift stability was significantly enhanced after compensation.

To analyze the impact of temperature compensation on TMR sensor accuracy, the concept of accuracy is introduced. Accuracy is defined as the degree of agreement between measured and true current values and is calculated as the percentage of the maximum deviation between the measured and true values over the full scale, as shown in Equation (17):

(17) $$\delta_{A}=\frac{\Delta I_{max}}{I_{FS}}\times 100\%.$$

Before temperature compensation, the maximum measurement error within the range of −10 mA to 10 mA was 1.399 mA at 120 °C, yielding a sensor accuracy of 6.99% FS. After temperature compensation, the maximum error was reduced to 0.056 mA at 90 °C, yielding an accuracy of 0.28% FS. The sensor’s measurement accuracy improved by 95.99%.

### 5.2. Continuous Temperature Variation Test

**Figure 12:**
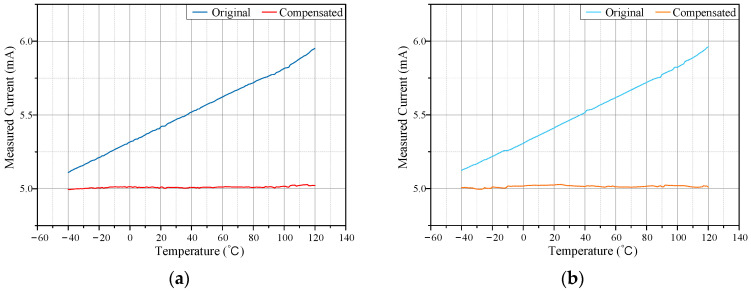
Current measurement of the continuous temperature-varying. (**a**) Heating process; (**b**) Cooling process.

This section evaluates the sensor’s performance under continuously varying environmental temperatures by testing its output in a temperature environment ranging from −40 °C to 120 °C. The temperature in the chamber was controlled to increase and decrease linearly using a programmed sequence. The test current was fixed at 5 mA, and measurements were taken while the temperature increased from −40 °C to 120 °C and decreased back to −40 °C. At the terminal temperatures, the chamber was held for one hour to stabilize before resuming testing. Figure 12a shows the sensor measurement results during these continuous temperature changes.

Figure 12b illustrates that, after temperature compensation, the sensor output remained stable around 5 mA during both the heating and cooling processes. In contrast, uncalibrated measurements showed significant errors as temperatures changed. Post compensation, the measurement error was less than 0.6%, enabling the high-precision measurement of power grid leakage currents in complex environments.

### 5.3. Alternating Current Test

**Figure 13:**
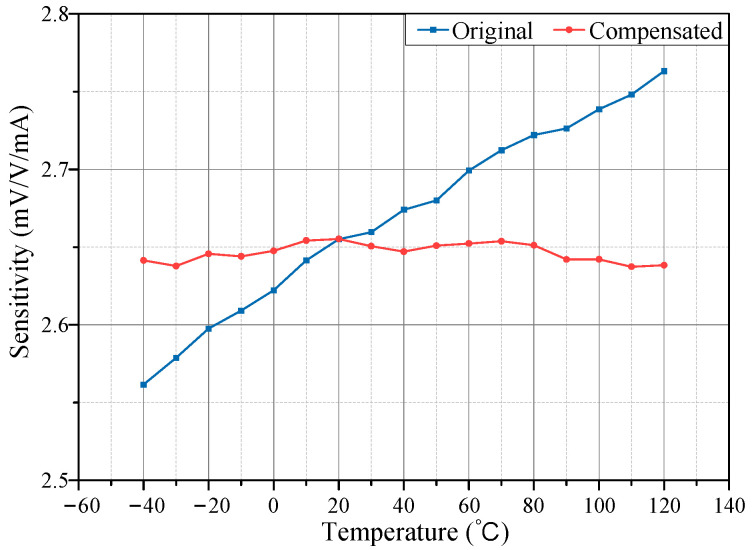
Comparison of sensor sensitivity before and after compensation when testing 50 Hz AC current.

The weak current sensor in this study is designed to measure leakage currents with a frequency range from DC to several tens of hertz. The response of the TMR current sensor to a 50 Hz AC current was tested. The test current was configured as a 50 Hz sine wave, with multiple test points selected in the range of 0–10 mA. Fast Fourier transform (FFT) analysis was performed to extract the 50 Hz signal. The temperature ranged from −40 °C to 120 °C, and sensor response testing was repeated. Figure 13 shows the changes in sensor sensitivity with temperature. The temperature coefficient of sensitivity (TCS) was calculated using Equation (16). Prior to compensation, the TCS was 474 ppm/°C; after compensation, it was reduced to 42 ppm/°C, representing a 91.14% decrease. These results indicate that, using the temperature compensation method presented, the TMR sensor accurately measured weak currents from DC to low frequencies over a wide temperature range.

### 5.4. Comparison with Other Methods

The proposed temperature compensation method for the TMR current sensor was compared with other methods from the literature, as summarized in Table 2. The proposed method achieved the lowest sensitivity temperature drift coefficient and effectively realized zero-offset temperature drift compensation, leading to the highest measurement accuracy over a wider temperature range. This approach addresses the temperature drift issue of TMR current sensors more effectively than previously reported methods.

## 6. Conclusions

This study addresses the temperature sensitivity of TMR current sensors in smart grids, particularly in weak current measurement scenarios, by proposing a high-precision software-based temperature compensation method implemented using FPGA. Through a systematic analysis of the working principles and temperature characteristics of TMR sensors, a compensation algorithm integrating cubic spline interpolation and polynomial regression is adopted, alongside a zero-offset self-calibration compensation circuit. An experimental platform was established to thoroughly evaluate the sensor’s performance across varying temperatures. The results indicate that the proposed compensation method significantly enhances the temperature stability of TMR sensors. Specifically, the sensitivity temperature drift coefficient was reduced from 1010 ppm/°C to 37 ppm/°C, representing a 96.34% improvement over a temperature range of −40 °C to 120 °C. Additionally, the zero-offset temperature drift was significantly reduced, improving zero-drift stability by 82.85%. The maximum measurement error within a ±10 mA range decreased from 1.399 mA to 0.056 mA, corresponding to a 95.99% improvement in accuracy. In simulations reflecting real-world temperature variations, the output for a 5 mA test current remained stable after compensation, demonstrating reliability and potential for practical application.

Compared to similar studies, the proposed method is effective over a wider temperature range and significantly compensates for both sensitivity and zero-offset drifts, thereby enhancing measurement accuracy. Overall, this research provides a solid foundation for the application of TMR sensors in smart grids, particularly for leakage current detection. Furthermore, the software-based temperature compensation method we propose is flexible, practical, highly adaptable, and easily portable. It can also be applied to other magnetoresistance sensors to mitigate temperature sensitivity. Additionally, our method simplifies the temperature calibration process when porting to other sensors, reducing the workload involved in calibration. Future research could explore more advanced algorithms, such as machine learning, to further enhance compensation accuracy and facilitate sensor–circuit integration, ultimately improving performance and reliability.

## Figures and Tables

**Table 1:** Comparison of errors for different degrees of polynomial regression.

Fitting Order	RMSE
First-order	6.055 × 10^−3^
Second-order	2.265 × 10^−3^
Third-order	2.261 × 10^−3^

**Table 2:** Comparison of different temperature compensation methods.

	[28]	[32]	[35]	[36]	This Work
Compensation Types	Hardware	Hardware	Software	Software	Software
Temperature Range	−10~60 °C	−40~80 °C	−45~85 °C	−20~80 °C	−40~120 °C
Pre-Compensation TCS	1780 ppm/°C	2498 ppm/°C	1100 ppm/°C	\	1010 ppm/°C
Compensated TCS	504 ppm/°C	678 ppm/°C	117 ppm/°C	\	37 ppm/°C
Measurement Precision	\	\	\	0.60%	0.28%

## Data Availability

Experimental data can be obtained by contacting the author via email (wujie221@mails.ucas.ac.cn).
